# A Model-Based Method for Detecting Persistent Cultural Change Using Panel Data

**DOI:** 10.15195/v8.a5

**Published:** 2021-03-22

**Authors:** Stephen Vaisey, Kevin Kiley

**Affiliations:** Duke University

**Keywords:** cultural change, personal culture, panel data, structural equation models, General Social Survey

## Abstract

Recent work argues that changes in people’s responses to the same question over time should be thought of as reflecting a fixed baseline subject to temporary local influences, rather than durable changes in response to new information. Distinguishing between these two individual-level process—a settled dispositions model and an active updating model—is important because these individual-level processes underlie different theories of population-level social change. This article introduces an alternative method for adjudicating between these two models based on structural equation modeling. This model provides a close fit to the theoretical models outlined in previous work. Applying this method to more than 500 questions in the General Social Survey’s three-wave panels, we find even stronger evidence than previous work that most survey responses reflect settled dispositions developed prior to adulthood.

Kiley and Vaisey (2020) recently published a method for assessing whether survey respondents appear to be changing their beliefs between waves or whether they instead appear to be repeating fixed responses with temporary local influences. This question is important because these processes reflect very different theoretical models of the evolution of “personal culture” (see Lizardo 2017). That is, if cultural beliefs are primarily public and responsive to external discourse, we should observe more updating as people respond to changes in their local environment. On the other hand, if cultural beliefs are primarily something learned early, then “settled dispositions” should be relatively resilient to change (see also Vaisey and Lizardo 2016).

In this article, we build on Kiley and Vaisey (2020) and introduce an alternative method for distinguishing between cases where respondents appear be actively updating their responses and situations where respondents’ responses appear to be settled. This method, based on structural equation modeling, provides a close fit to the theoretical models outlined in Kiley and Vaisey (2020) and provides even stronger support for their claim that most cultural beliefs reflect settled dispositions developed prior to adulthood.

## Background

**Figure 1:**
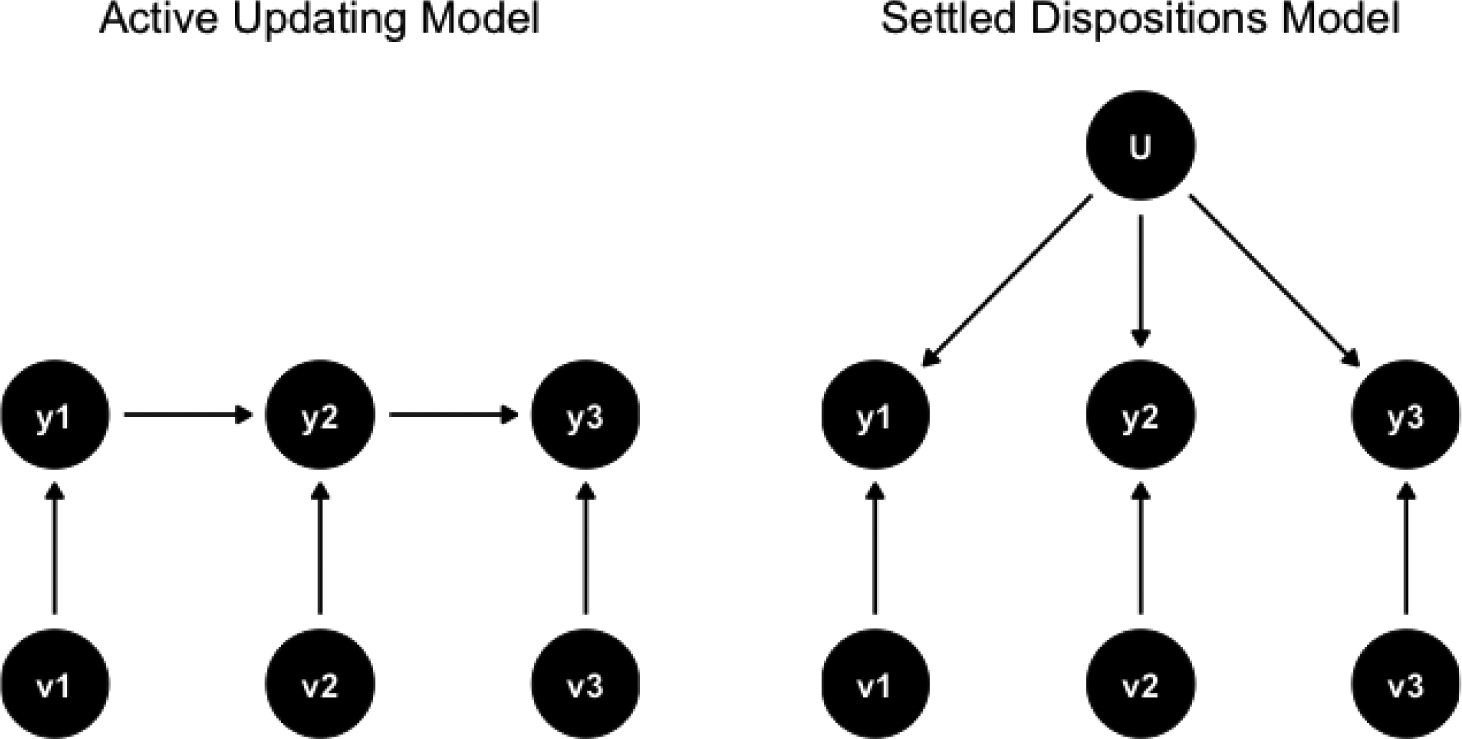
Theoretical models.

Kiley and Vaisey (2020; hereafter KV) distinguish between two main models of cultural change implied in the literature: the *active updating model* (AUM) and the *settled dispositions model* (SDM). The assumptions of each model are represented in Figure 1. The active updating model assumes that shocks affecting a person’s response to a question at one point in time persist to the next time period, whereas the settled dispositions model assumes that shocks do not persist.

KV’s goal is to develop a method that can distinguish between the two processes. They proceed by estimating the following equation on three-wave panel data:

(1) $$E\left(y_{i3}\right)=\alpha +\phi \beta y_{i2}+(1-\phi )\beta y_{i1}.$$

The intuition behind Equation (1) is that, if people are updating their beliefs, survey responses that are closer in time (e.g., wave 1 and wave 2) should be more similar than responses that are more distant in time (wave 1 and wave 3). The *ϕ* parameter indicates the relative weight given to the more proximate wave 2 response (*ϕ*) and the more distal wave 1 response (1 − *ϕ*). As *ϕ* approaches 1, wave 2 becomes more informative about wave 3, and wave 1 becomes less informative about wave 3, suggesting persistent change between waves. As *ϕ* approaches 0.5, however, both prior waves are assigned equal weight, suggesting that the answers reflect a persistent disposition to answer the question in a particular way.^1^

Estimating this model on 183 variables from the 2006 to 2014 panels of the General Social Survey (GSS), KV find that about 40 percent of the variables show no evidence of active updating, and most of the remainder show only weak evidence of active updating. Although they point out several exceptions (e.g., some questions about gay rights, where attitudes do appear to be changing over the study period) they conclude that the settled dispositions model is a better default for thinking about public opinion among adults.

## An Alternative Method

### From Theoretical to Statistical Models

The alternative method we propose uses structural equation modeling to implement the theoretical graphs more directly. By estimating the model this way, we can compare a wide variety of model specifications that have different theoretical implications (see Bollen and Brand 2010). For example, we could specify the core graph of the settled dispositions model as follows:

(2) $$y_{i1}=\alpha_{1}+U_{i}+\epsilon_{i1},\;y_{i2}=\alpha_{2}+U_{i}+\epsilon_{i2},\;y_{i3}=\alpha_{3}+U_{i}+\epsilon_{i3}.$$

From this basic version, we can add further constraints. For example, we can constrain *α*_1_ = *α*_2_ = *α*_3_ if we are willing to assume that the mean response is the same at all waves. And we can constrain $\sigma_{\epsilon_{1}}^{2}=\sigma_{\epsilon_{2}}^{2}=\sigma_{\epsilon_{3}}^{2}$ if we are willing to assume that the variance of the responses does not change over time.

This is not the place for a general overview of structural equation modeling (Bollen 1989; see Kline 2015 for an accessible introduction). But the basic intuition in the three-wave case is that we are attempting to reconstruct nine observed elements from the data (three means, three variances, and three covariances) using a simpler model with fewer than nine parameters. For example, if we make the assumptions in Equation (2), including the equality constraints in the following paragraph, we can estimate a model that has only three parameters: one value of *α* (shared by all waves), one $\sigma_{\epsilon }^{2}$ (shared by all waves), and one $\sigma_{U}^{2}$. Here *U* is a “latent variable,” which, in this specification, simply means a person-specific “error” (or fixed effect) that gets added equally to a person’s response at every wave. *U*_*i*_ reflects an individual’s unchanging tendency to respond to a certain question the same way over the study period (a “settled disposition”).

Using this model will not work well to reconstruct the observed data if the data were generated by a process that looks like the active updating model (on the left of Figure 1). We could instead estimate the following model:

(3) $$y_{i3}=\alpha_{3}+\rho y_{i2}+\epsilon_{i3},\;y_{i2}=\alpha_{2}+\rho y_{i1}+\epsilon_{i2}.$$

Here we cannot make *Y*_1_ a dependent variable because we don’t have its previous value (which would be *Y*_0_). Therefore we can only use *Y*_1_ as a predictor. As above, we can make further simplifications if we want (e.g., *α*_2_ = *α*_3_ or $\sigma_{\epsilon_{2}}^{2}=\sigma_{\epsilon_{3}}^{2}$). Using this fully simplified version, we are estimating a model with five parameters: one value of *α* (shared by waves 2 and 3), one $\sigma_{\epsilon }^{2}$ (shared by waves 2 and 3), the *ρ* autocorrelation parameter, and the mean and variance of *Y*_1_.

This model constrains *ρ* to be equal across waves. This implies that the rate of change between the two waves is equal, which comes with two implications. First, it assumes that the time between waves is equal. If waves are differently spaced, more time between waves is likely to equate to more active updating. This assumption is reasonable for the GSS panels, where surveys took place about two years apart.^2^ This might not be a reasonable assumption in other panels and can be loosened by adding a coefficient to *ρ* for the time since the last wave.

Second, constraining *ρ* to be the same across waves implies that active updating happens at a constant rate. Although this is likely a valid assumption for many questions we explore in the GSS, there are questions where opinions likely change discontinuously and in durable ways around specific dates and events. In the GSS panels, these questions include confidence in various branches of the government, which might change as a result of elections or high-profile political events, and confidence in financial institutions, belief in the ability to find a job, and subjective socioeconomic status, which might change in response to the onset of the Great Recession.^3^

In our framework, *ρ* should be thought of as a detector of whether any active updating happens in the panel window, rather than as a measure of the rate of active updating over time. If any updating happens in the panel window, *ρ* will tend to be nonzero. However, we cannot rule out the possibility that all attitudes could demonstrate durable updating under certain circumstances, only whether they demonstrate updating in our study window. We return to this point in the discussion below.

If *Y*_*t*_ were always measured perfectly, we could simply compare the penalized likelihoods (e.g., Bayesian information criteria) of the two models to see which is more likely to be the true model given the data (Bollen et al. 2014). Unfortunately, most GSS items likely contain some measurement error. Any measurement bias—the systematic tendency for an item to over- or underestimate a person’s “true” value—will induce error correlations between waves that have no basis in the causal process. Thus *U*_*i*_ in Equation (2) represents both settled dispositions and respondent-specific measurement bias.

For this reason, the most reasonable specification of the active updating model combines features of Equations (2) and (3), as follows:

(4) $$y_{i3}=\alpha_{3}+\rho y_{i2}+U_{i}+\epsilon_{i3},\;y_{i2}=\alpha_{2}+\rho y_{i1}+U_{i}+\epsilon_{i2},\;\text{Cov}\left(U,Y_{1}\right)=\tau .$$

Adding *U* to Equation (4) allows for systematic correlations between the wave 2 and wave 3 responses due to measurement bias or error.^4^ Estimating *τ*, the covariance between *U* and *Y*_1_, reflects that *U* and *Y*_1_ share common unobserved causes (e.g., measurement bias, values of the presurvey *Y*_0_).

**Figure 2:**
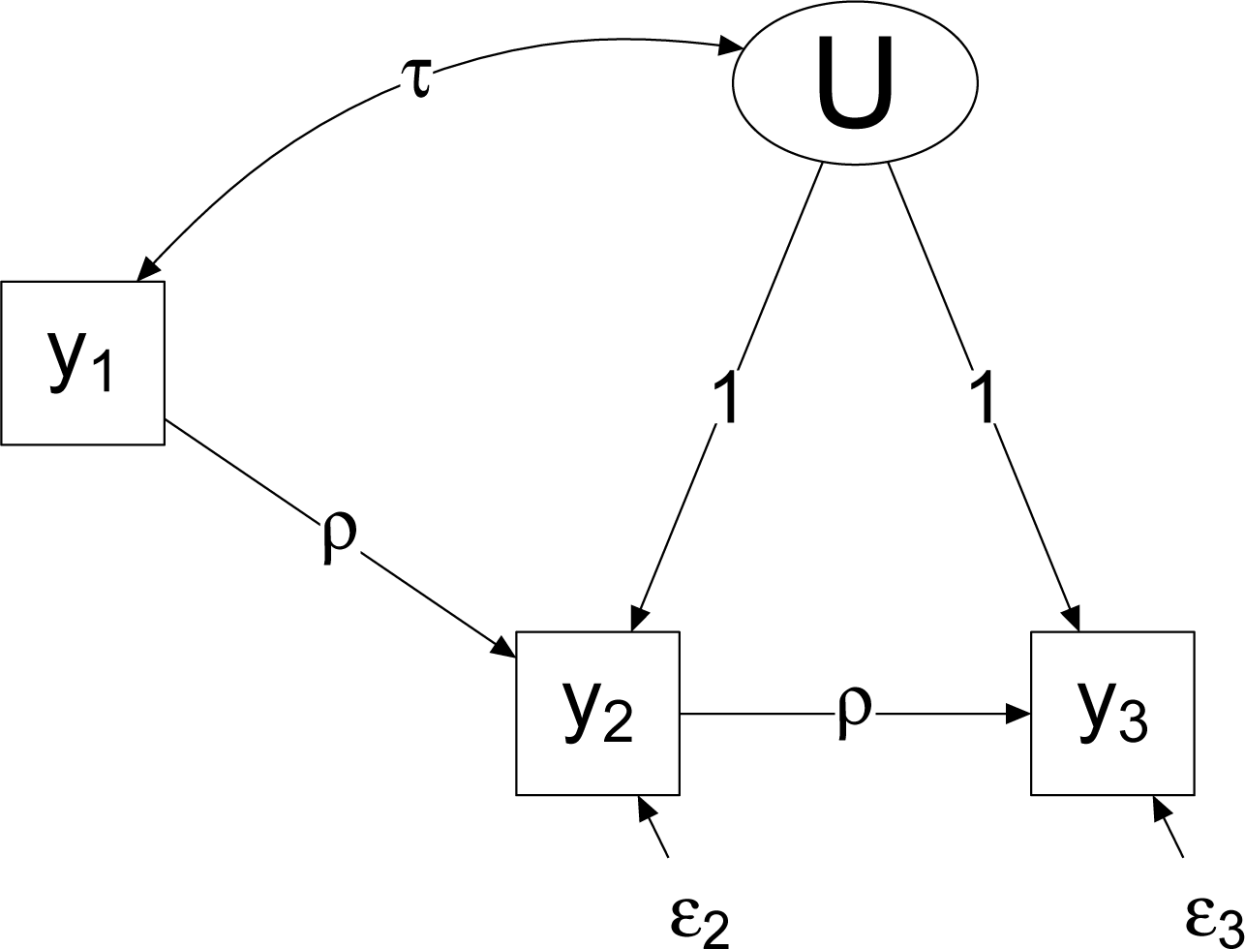
General model.

The most straightforward^5^ way to compare models is to specify them as special cases of a more general model. Figure 2 shows the basic model.

We have three basic choices to make:

1. Do we allow wave-to-wave updating of responses (estimate *ρ*)?
2. Do we allow for settled dispositions and systematic bias (estimate $\sigma_{U}^{2}$ and *τ*)?
3. Do we allow wave-to-wave changes in the mean response (estimate *α*_2_ and *α*_3_ separately) or assume no aggregate change (*α*_2_ = *α*_3_)?

Crossing these three binary choices would normally lead to eight candidate models. However, we exclude from consideration models that contain neither updating nor settled dispositions because no theoretical perspective argues for them. This leaves us with six models, shown in Table 1.

For any given three-wave panel, we can compare the fit of these models against each other to help determine which of the models is most likely to have generated the data.

### Advantages of the Approach

Kiley and Vaisey (2020) also compare estimates using Equation (1) with and without constraints to adjudicate between the active updating and settled dispositions accounts. The approach we outline here is the same in spirit but different in the details. The main difference is that KV compare models based solely on how well they predict the wave 3 response. Using structural equation models allows comparing the fit of the model to *all* the observed data, not just the final response. For example, the KV model has no leverage in cases where respondents give the same response in waves 1 and 2 but a different response in wave 3, whereas the model presented here does.

To oversimplify slightly, the models presented here essentially adjudicate whether people are equally likely to deviate from their baseline response in all waves, which would be evidence of the settled dispositions model, or whether waves 1 and 3 are more likely than wave 2 to be deviations, which would be evidence of persisting changes and active updating.

An additional advantage of this approach is that it can be more easily extended to panel data sets that include a larger number of waves than the model presented in KV. Although three waves is the minimum number required to adjudicate the two theoretical approaches outlined above, and what we focus on in this article, adding more waves can help adjudicate the two theoretical models under a wider array of assumptions.

### Analytic Strategy

We use the same 183 variables and three panels of the General Social Survey as Kiley and Vaisey (2020). Rather than pool the panels as they do, we estimate models separately on each one. Most variables appear in all three panels, but six are measured in only one panel, and one is measured in two panels. This gives us 536 total three-wave data sets.

For each of the 536 data sets, we estimate all six of the candidate models. We then compare fits using the Bayesian information criterion (BIC; see Raftery 1995; Bollen et al. 2014). In order to make the best case possible for each theoretical model, we will compare the best fitting of the four AUM models with the best fitting of the two SDM models. In cases where the BIC difference between the two “finalist” models is less than two, we consider the evidence inconclusive (Raftery 1995; Bollen et al. 2014).

The approach is quite conservative, favoring the active updating model over the settled dispositions model when there is meaningful evidence that some amount of people in the sample are making durable changes in opinion or behavior. This should not be taken as evidence that *many* people in the population are making durable changes, only that there is any evidence of durable change at all.

## Results

### BIC Comparisons

**Figure 3:**
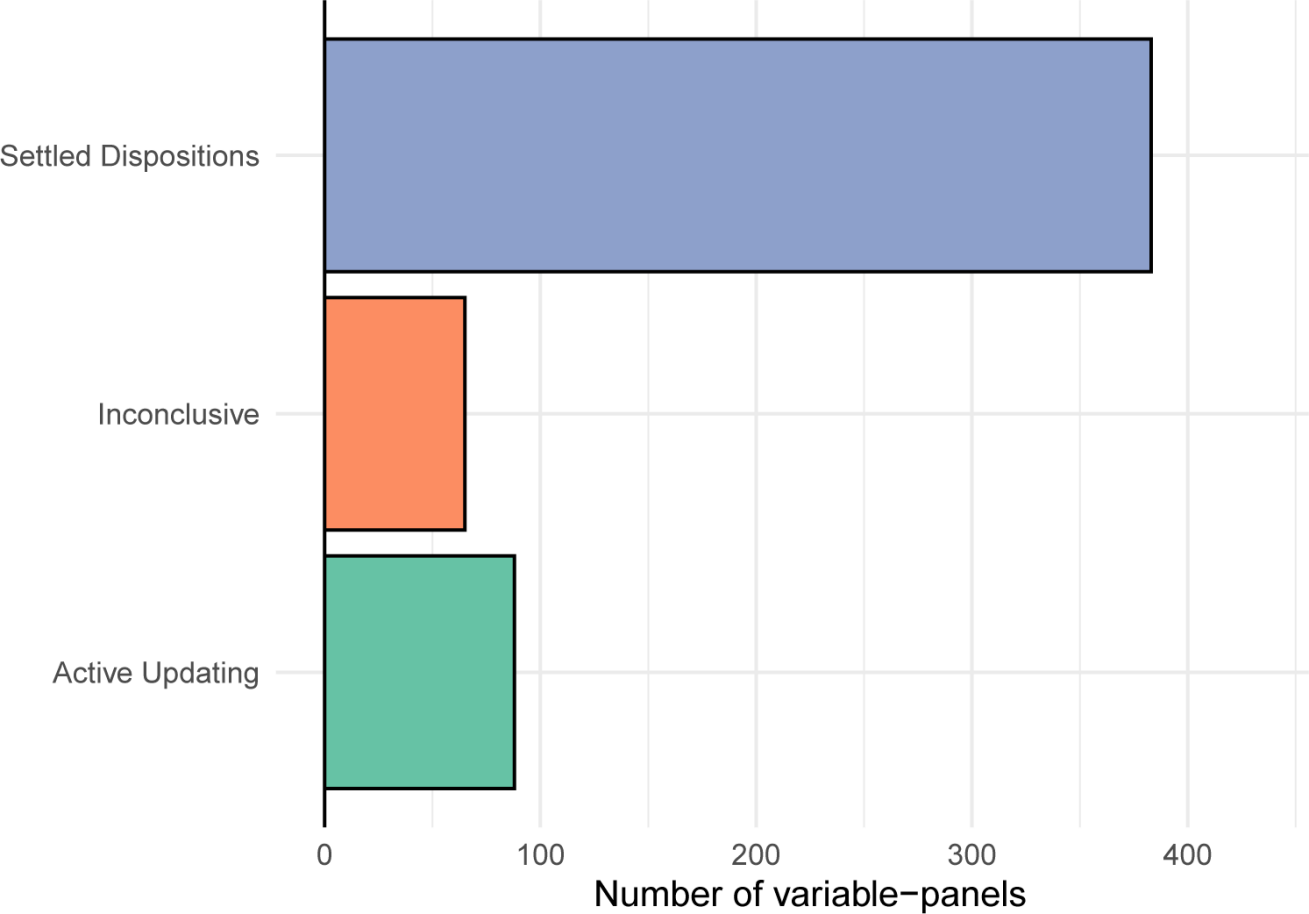
Count of preferred models, determined through BIC comparisons. Comparisons where BIC difference between best-fitting SDM and best-fitting AUM was less than two were classified as inconclusive.

Figure 3 shows the results of these comparisons for all 536 variable-panels. One of the two SDM models is preferred for 71 percent of cases, with one of the four AUM models preferred in 16 percent of cases. The remaining cases are inconclusive.

If we look at only variables measured in all three panels, we can also compare the number of variables where all three agree. There are 69 variables that unanimously point to the SDM and only three that unanimously favor the AUM. The three unanimous AUM variables are news (reading a newspaper), owngun (having a gun in your home), and socbar (going to a bar or tavern). The presence of owngun on this list is promising because we *know* that physical objects act according to an active updating model—when you buy a gun, it stays in your house until you get rid of it.

### Goodness of Fit

Almost all (99.5 percent) of the preferred SDM models have acceptable fits (root mean square error of approximagion [RMSEA] < 0.08). But we can put the SDM to an even stronger test by estimating a model that assumes no updating across waves *and* no changes in the means (i.e., no period effects). This is the model implied by Equation (2) and is equivalent to a simple confirmatory factor analysis with just three parameters: one value of *α* (shared by all waves), one $\sigma_{\epsilon }^{2}$ (shared by all waves), and one $\sigma_{U}^{2}$. In essence, this model assumes that each wave is a report of a fixed quantity determined before the time of the study.

**Figure 4:**
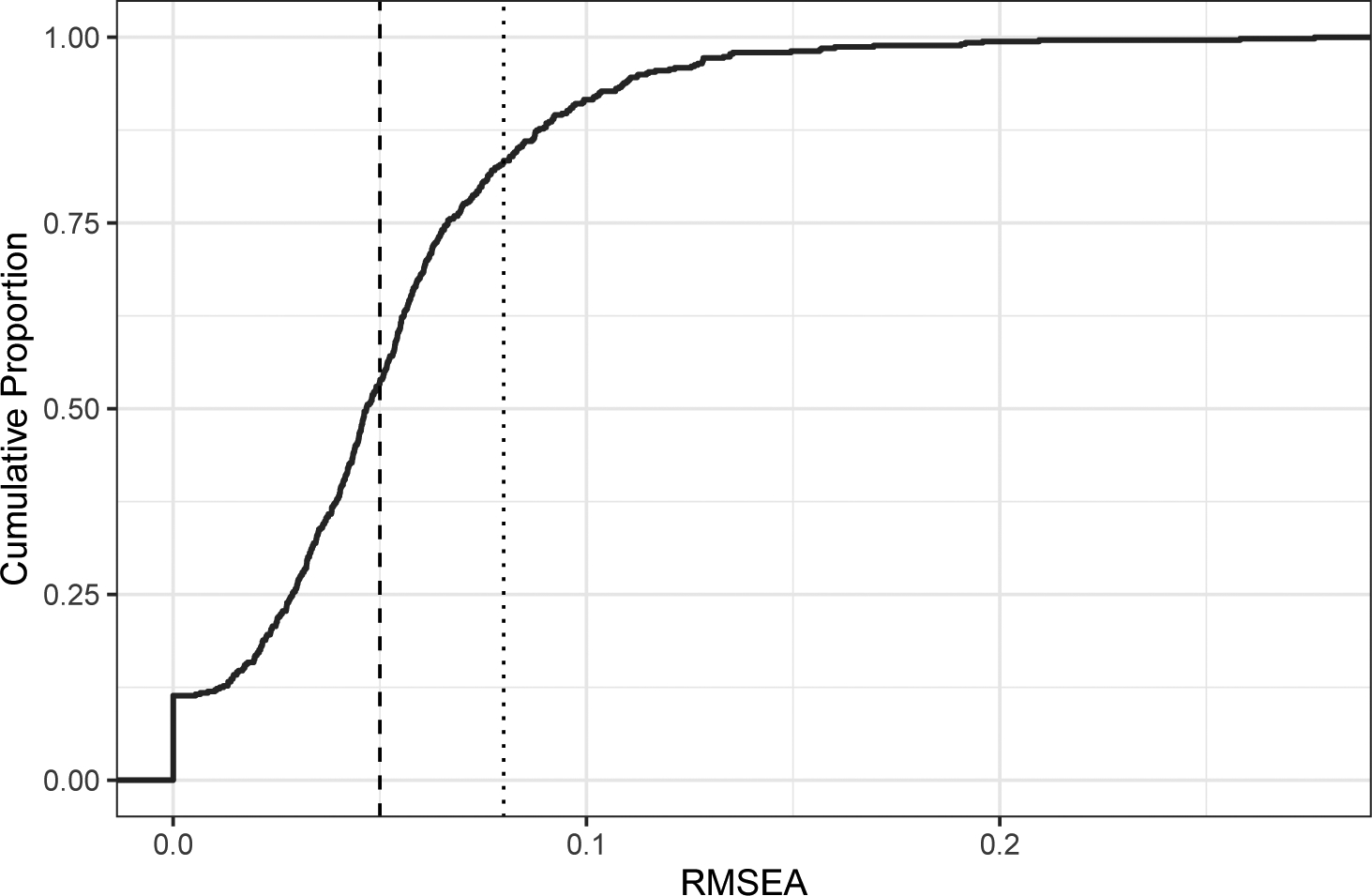
Distribution of fit values for simple confirmatory factor analysis.

Estimating this model shows that it fits 83.4 percent of the variable-panels acceptably (RMSEA < 0.08) and 53.4 percent of the variable-panels well (RMSEA < 0.05). Figure 4 shows the overall distribution of RMSEA values. We don’t want to take these conventional cutoffs too literally (see Barrett 2007), but altogether these results indicate that the majority of GSS items are highly compatible with the settled dispositions model.

## Discussion

Building on Kiley and Vaisey (2020), we have presented a model-based approach to estimating whether the pattern of attitude and behavior change in the population should be best thought of as following a *settled dispositions* model, where people have stable baselines and change (if any) is temporary, or an *active updating* model, where changes tend to persist. Using a set of structural equation models that formalize these theoretical processes under a variety of assumptions, we compared the best-fitting settled dispositions model with the best-fitting active updating model for each variable in each panel, a total of 536 variable-panel pairs. The overwhelming majority of variable-panels prefer the settled dispositions model, with 69 variables preferring the settled dispositions model in all panels and only three variables preferring the active updating model in all panels.

Although we cannot account for attitudes and behaviors that are not measured here, these results suggest, consistent with KV, that a wide range of attitudes and behaviors tend to be settled by the time people are old enough to participate in surveys like the GSS. Although this is not surprising because we examine the same set of questions as KV, it is important that different statistical assumptions lead to similar substantive conclusions.

It is important to note here that settled opinions does not necessarily mean stable (unchanging) opinions. People might change their responses from wave to wave, but the settled dispositions model assumes this change does not tend to persist, meaning it reflects temporary changes in opinion or measurement error.^6^ There is little evidence of even a moderate amount of durable change in opinions in the medium term (two to four years). Perhaps even more striking, the results in the section on goodness of fit show that the majority of variables are consistent with no population-level change in attitudes or behaviors at all over the four-year period.

As noted above, the fact that most attitudes prefer the settled dispositions model in the panel window should not be taken to imply that settled attitudes cannot durably update if circumstances change. Consistent with previous work, the results presented here suggest that circumstances occasionally change and lead to durable attitude change. At the same time, our results suggest that the kinds of circumstantial change that lead to durable attitude change seem relatively rare and seem confined to major public events. If local changes such as changes in social network composition do lead to durable updating in attitudes, these local changes are so rare as to be undetectable on most issues measured here.

On specific variables, the approach presented here occasionally suggests different conclusions than the approach presented in KV, as would be expected under models that make different assumptions, with the model presented here favoring the SDM model in more cases. There are two main reasons for these differences. First, KV pool their panels together, and the method favors the active updating model if any updating is present, meaning that if *any* of the three panels show updating, the variable as a whole is likely to show updating. A second reason these differences emerge is that the model presented here uses slightly more data than the KV model. A person who gives the same response in waves 1 and 2 but a different response in wave 3 is mostly useless under the KV model but can factor into the calculation in the model presented here. Because variable-panels can switch from favoring the active updating model to favoring the settled dispositions model with the addition or subtraction of a few cases, this suggests that evidence of the AUM is very weak even when it is favored. In particular, when the AUM is favored, it is often because a very small proportion of the population is making durable changes. It is difficult to draw broader conclusions about these discrepancies, as they cut across both question substance and question structure.

**Figure 5:**
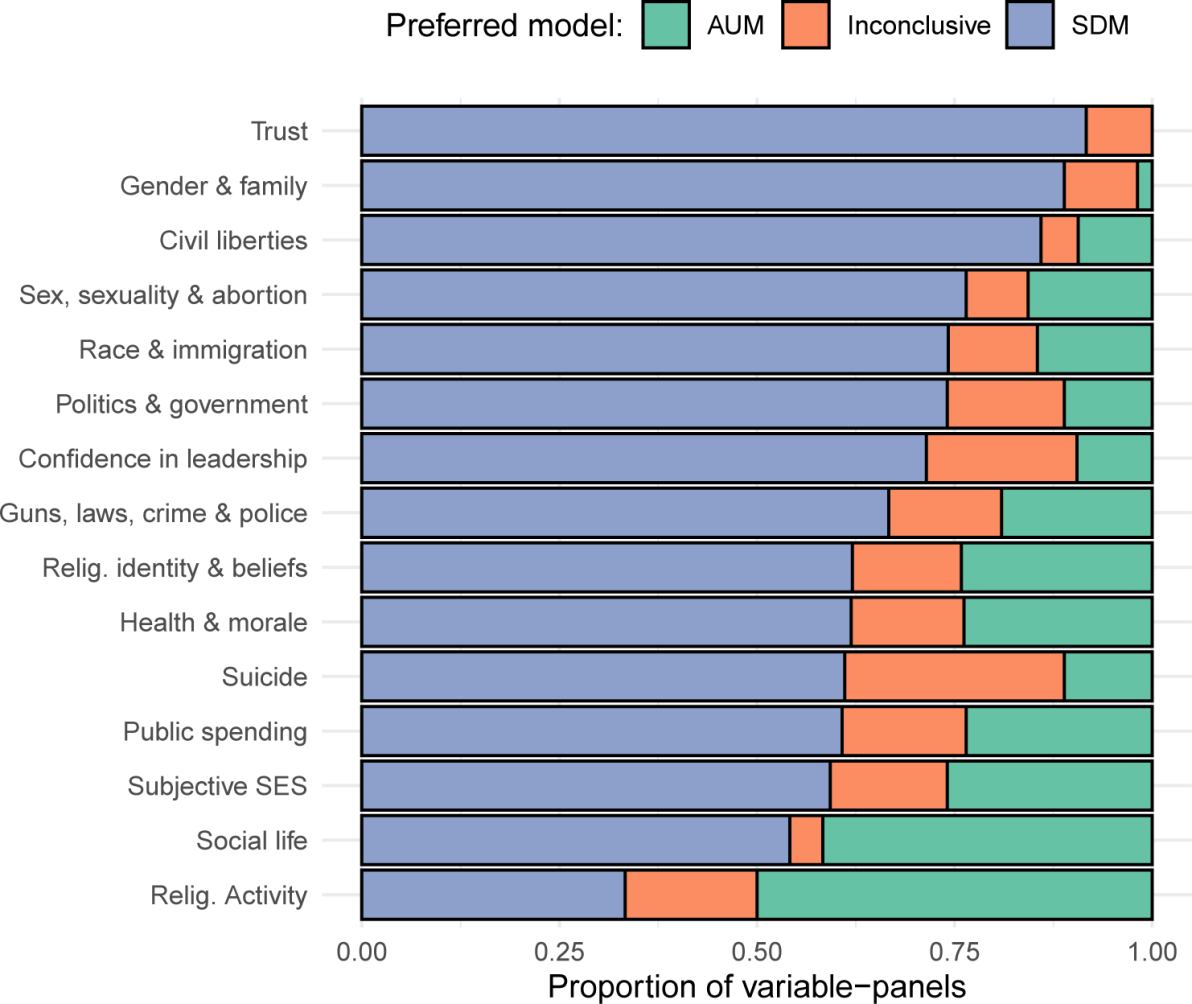
Proportion of variable-panels preferred by each model, by subject category. SES, socioeconomic status.

Although it would be impossible to discuss all 183 variables or 536 variable-panels in depth, there are some broad patterns worth highlighting. Figure 5 plots the proportion of variable-panels preferring each model by subject material.^7^ KV’s results suggest that issues related to civil liberties, gender, and social trust tend to be settled by the time people entered the the GSS. The method outlined above finds similar results, with 86 percent, 89 percent, and 91 percent of variable-panels in these categories, respectively, favoring the settled dispositions models. These are the three categories with the largest proportions of variable-panels favoring the settled dispositions model.

Similarly, KV suggested that public behaviors were more likely to demonstrate active updating because these behaviors receive social reinforcement that facilitates durable change. In the results presented here, social behaviors such as socializing at a bar and religious behaviors such as church attendance are the two categories that show the highest rates of active updating, with 41 percent and 50 percent of variable-panels in these categories, respectively, showing evidence of active updating.

Another issue worth touching on is variation across panels in favoring the settled dispositions or active updating models within the same question. Of the 176 questions that appear in all three panels, 110 either favor the settled dispositions model or are inconclusive in all three waves (with 69 clearly favoring active updating in all panels). However, 58 variables favor each model in at least one panel.

Some of these discrepancies are likely due to real changes in the social environment that only affect members of one panel. For example, several of the questions with divergent findings pertain to whether the federal government is spending too little, about right, or too much on various priorities. People’s responses might be changing in response to changes in federal spending, changes in who controls the federal government, or changes in public messaging around these issues, which might only happen in certain years.

For questions that demonstrate active updating in at least one panel but have no obvious changing external referent (such as church attendance, religious activity, partisan identification, frequency of prayer, and support for marijuana legalization), durable change might be happening but occur at such low rates that finite samples using imperfect measures do not capture enough respondents undergoing durable change in a particular panel to favor the active updating model. Again, even on generally stable issues we expect some people in the population to be actively updating their behavior—most people know an adult who has changed their opinion on some issue—but this should be thought of as the exception.^8^ Our results suggest that when we observe someone change their response to a survey question, the assumption should be that it will not be a permanent change.

This points to a limitation of both this approach and the one used in Kiley and Vaisey (2020). Neither approach can quantify the proportion of the sample that is undergoing active updating. It is not clear whether, for the variable-panels that show evidence active updating, there is a large or small proportion of respondents making durable changes.

The two approaches share additional limitations. Both approaches assume linearity in the variable being measured. If an outcome is measured on an ordinal scale such as a four-point Likert scale (strongly agree, agree, disagree, strongly disagree), a change from agree to strongly agree is treated the same as a change from disagree to agree, which might or might not be a reasonable assumption. Both approaches also rely on weights to account for differential nonresponse over the course of the panel. If the mechanisms of nonresponse and active updating are both driven by some unobserved variable, then our results might underestimate the probability of durable change. That being said, we have no reason to assume that nonresponse is more likely to be related to active updating than settled dispositions.

## Conclusion

The approach presented here and the approach Kiley and Vaisey (2020) use attempt to adjudicate a similar theoretical question: are there more people reporting patterns that look like active updating than we would expect if people behaved according to a settled dispositions model? The difference between the two approaches are sets of assumptions about what counts as an active updating pattern and what counts as a settled dispositions pattern. The fact that different sets of assumptions reach the same substantive conclusion, even under tests very favorable to detecting active updating, strengthens the findings from KV and others (e.g., Vaisey and Lizardo 2016) that the settled dispositions model should be thought of as the dominant model for attitude behavior in adults.

These findings have clear implications for cultural sociology and public opinion research, favoring models of cultural change rooted in cohort replacement (Mannheim 1970; Ryder 1965) and those that place importance on early-life socialization over contemporary social environments (such as Bourdieu 1990). We believe that these findings are also relevant for research on cultural evolution (see Henrich 2015; Heyes 2018; Mesoudi 2016) because they place significant limits on the likely pace and mechanisms of cultural change. Cohort-based change will necessarily be slower than that implied by simple population models of social learning. This lag process allows cultural equilibria to be durable even when the contemporary information environment is changing (O’Connor 2019). We hope future research explores these links more directly.

## Notes

1. The *β* parameter reflects the extent to which wave 3 is predictable from *any* combination of wave 1 and wave 2. Questions with low *β* are simply “noisy.”
2. Across the three panels, the average time between waves 1 and 2 is between 695 and 764 days, whereas the average time between waves 2 and 3 is between 692 and 743 days.
3. Despite the (potentially erroneous) assumption of linear updating, these questions still prefer the active updating model in relevant windows in the analysis below.
4. Unfortunately, in the three-wave case there’s no way to distinguish systematic measurement bias or error from true person-level stable differences as components of *U* without additional untestable assumptions.
5. We could specify an even more parsimonious version of the settled dispositions model by estimating Equation (2) with the equality assumptions discussed in the text. This would effectively be estimating a confirmatory factor analysis on *Y*_*t*_. This would only require three parameters. We will revisit this in the section on goodness of fit.
6. Within the settled dispositions model, our method cannot distinguish between change that occurs because people lack opinions and are more or less guessing at random (Converse 1964), change that occurs because people are influenced by short-term messaging and other considerations (Zaller 1992), or changes that occurs because of other forms of measurement error (Ansolabehere, Rodden, and Snyder 2008; Alwin 2007). We assume that these short-term changes are some combination of these forces, which might vary by question.
7. It should be kept in mind when comparing across subject areas that the questions in subject areas often have different structures, and the questions asked in each category should not be thought of as a random sample of issues in that domain.
8. One reason people can recall examples of people in their social networks who have changed beliefs and behaviors is that it is rare and tends to stick out.

## Figures and Tables

**Table 1:** Candidate models and their parameter constraints.

Model	$\sigma_{U}^{2},\;\tau$	*ρ*	*α*_*t*_	# parameters
AUM1	0	free	=	5
AUM2	0	free	free	6
AUM3	free	free	=	7
AUM4	free	free	free	8
SDM1	free	0	=	6
SDM2	free	0	free	7
